# Supplementary material for: Oral Administration of a Phage Cocktail to Reduce Salmonella Colonization in Broiler Gastrointestinal Tract—A Pilot Study
Source: Animals (Basel). 2022 Nov 9;12(22):3087. doi: 10.3390/ani12223087 (PMC9686501; doi:10.3390/ani12223087)
Supplement: Supplementary file 1 [file animals-12-03087-s001.zip › animals-1963554-supplementary.pdf]

Table S1. Serovars of *Salmonella* used for host-range determination

**Serovars**	**Codename**	**Source of origin**[1]	**Antigenic determinants**		
			O antigen	H phase I	H phase II
Agona	H3D6	CBF	1,4,12	f,g,s	[1,2]
Albany	H32	CBF	8,20	z_4,z_{23}	-
Corvallis	F3-W5-S2	CBF	8,20	z_4,z_{23}	[z_6]
Kentucky	S1H28	CBF	8,20	i	z_6
Mbandaka	H17D2	CBF	6,7,14	z_{10}	e,n,z_{15}
Saintpaul	H13	CBF	1,4,[5],12	e,h	1,2
Schwarzengrund	H2	CBF	1,4,12,27	d	1,7
Typhimurium	S5-370	Human (US)	1,4,[5],12	i	1,2
Anatum	A4-525	Bovine (US)	3,10[15][15,34]	e,h	1,6
Braenderup	S5-373	Human (US)	6,7,14	e,h	e,n,z_{15}
Cerro	R8-242	Laboratory strain	6,14,18	z_4,z_{23}	[1,5]
Dublin	W1-016	Dairy farm (TH)	1,9,12[Vi]	g,p	-
Enteritidis	S5-371	Human (US)	1,9,12	g,m	-
Give	H2-018	Dairy farm (TH)	3,10[15][15,34]	[d],l,v	1,7
Hadar	PPI-013	Laboratory strain	6,8	z_{10}	e,n,x
Heidelberg	S5-455	Human (US)	1,4,[5],12	r	1,2
Infantis	S5-372	Laboratory strain	6,7,14	r	1,5
Javiana	S5-406	Human (US)	1,9,12	l,z_{28}	1,5
Kedougou	H2-061	Pig slaughterhouse (TH)	1,13,23	i	l,w
Montevideo	S5-403	Bovine (US)	6,7,14	g,m,[p],s	[1,2,7]
Muenster	S5-917	Bovine (US)	3,10[15][15,34]	e,h	1,5
Newport	S5-515	Laboratory strain	6,8,20	e,h	1,2:[z_{67}]
Oranienburg	R8-376	Human (US)	6,7,14	m,t	[z_{57}]
Panama	S5-454	Human (US)	1,9,12	l,v	1,5
Paratyphi B	H2-076	Laboratory strain	1,4,[5],12	b	1,2
Rissen	H2-015	Pig slaughterhouse (TH)	6,7,14	f,g	-
Singapore	PSU-BS-174SL	Food processing plant	6,7	k	e,n,x
Stanley	S5-464	Human (US)	1,4,[5],12,27	d	1,2

Table S1. Serovars of *Salmonella* used for host-range determination (continue)

Serovars	Codename	Source of origin	Antigenic determinants		
			O antigen	H phase I	H phase II
Aberdeen	DMST15678	Standard DMST	11	i	1,2
Abony	DMST21863	Standard DMST	1,4,[5],12,27	b	e,n,x
Alachua	DMST10896	Standard DMST	35	z_4,z_{23}	-
Amsterdam	DMST24706	Standard DMST	3,10[15][15,34]	g,m,s	-
Bangkok	DMST7121	Standard DMST	38	z_4,z_{24}	-
Bergen	DMST10895	Standard DMST	47	i	e,n,z_{15}
Blockley	DMST16821	Standard DMST	6,8	k	1,5
Choleraesuis	DMST8014	Standard DMST	6,7	c	1,5
Derby	DMST8353	Standard DMST	1,4,[5],12	f,g	[1,2]
Gallinarum	DMST15968	Standard DMST	1,9,12		-
Havana	DMST7116	Standard DMST	1,13,23	f,g,[s]	-
Hvittingfoss	DMST15681	Standard DMST	16	b	e,n,x
Krefeld	PSU-BS-111SL	Food processing plant	1,3,19	y	l,w
London	DMST7110	Standard DMST	3,10[15]	l,v	1,6
Ondersterpoort	DMST15680	Standard DMST	1,6,14,[25]	e,h	1,5
Ordonez	PSU-BS-181SL	Food processing plant	1,13,23	y	l,w
Paratyphi A	DMST8486	Standard DMST	1,2,12	a	[1,5]
Poona	DMST15679	Standard DMST	1,13,22	z	1,6
Senftenberg	DMST17013	Standard DMST	1,3,19	g,[s],t	-
Tennessee	PSU-BS-183SL	Food processing plant	6,7,14	Z_{29}	[1,2,7]
Typhi	DMST22842	Standard DMST	9,12[Vi]	d	-
Urbana	DMST10891	Standard DMST	30	b	e,n,x
Virginia	DMST17373	Standard DMST	8	d	1,2
Waycross	DMST19205	Standard DMST	41	z4,z23	[e,n,z_{15}]
Virchow	H2-117	Human (US)	6,7	r	1,2
Wandsworth	H2-068	Laboratory strain	39	b	1,2
Weltevreden	R8-798	Human (US)	3,10[15]	r	z_6
4,5,12: i: -	S5-390	Laboratory strain	4,5,12	i	-

[1] CBF: commercial broiler farm; Standard DMST: standard strains received from the Department of Medical Sciences, Ministry of Public Health, Thailand. Strains were also received from different sources from the United States (US) or Thailand (TH).
